# A Computer Vision-Based Roadside Occupation Surveillance System for Intelligent Transport in Smart Cities

**DOI:** 10.3390/s19081796

**Published:** 2019-04-15

**Authors:** George To Sum Ho, Yung Po Tsang, Chun Ho Wu, Wai Hung Wong, King Lun Choy

**Affiliations:** 1 Department of Supply Chain and Information Management, The Hang Seng University of Hong Kong, Shatin, Hong Kong, China; georgeho@hsu.edu.hk (G.T.S.H.); collinwong@hsu.edu.hk (W.H.W.); 2 Department of Industrial and Systems Engineering, The Hong Kong Polytechnic University, Hunghom, Hong Kong, China; p.tsang@connect.polyu.hk (Y.P.T.); kl.choy@polyu.edu.hk (K.L.C.)

**Keywords:** smart mobility, computer vision, roadside occupation, traffic surveillance, smart city

## Abstract

In digital and green city initiatives, smart mobility is a key aspect of developing smart cities and it is important for built-up areas worldwide. Double-parking and busy roadside activities such as frequent loading and unloading of trucks, have a negative impact on traffic situations, especially in cities with high transportation density. Hence, a real-time internet of things (IoT)-based system for surveillance of roadside loading and unloading bays is needed. In this paper, a fully integrated solution is developed by equipping high-definition smart cameras with wireless communication for traffic surveillance. Henceforth, this system is referred to as a computer vision-based roadside occupation surveillance system (CVROSS). Through a vision-based network, real-time roadside traffic images, such as images of loading or unloading activities, are captured automatically. By making use of the collected data, decision support on roadside occupancy and vacancy can be evaluated by means of fuzzy logic and visualized for users, thus enhancing the transparency of roadside activities. The CVROSS was designed and tested in Hong Kong to validate the accuracy of parking-gap estimation and system performance, aiming at facilitating traffic and fleet management for smart mobility.

## 1. Introduction

Traffic congestion is a persistent problem worldwide, leading to economic and social challenges. To enhance competitiveness, smooth traffic conditions are of the utmost importance for any city. This is especially true when developing a smart city, which aims at making good use of information and communication technologies (ICT) to support the creation of a ubiquitous and interconnected network of citizens and organizations, sharing digital data and information via the internet of things (IoT) [1]. In recent years, smart mobility and smart transportation have been advocated to alleviate the above social and public problems. 

**Figure 1:**
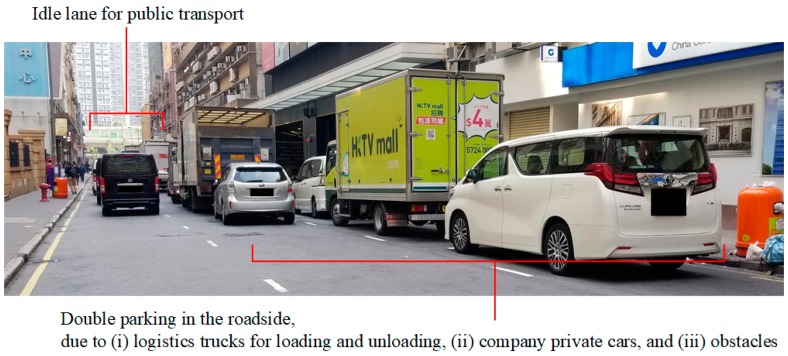
On-site roadside example of double-parking.

In general, frequent roadside loading and unloading activities have a negative impact on traffic situations. An increasing number of vehicles and insufficient data transparency regarding roadside activities, occupancy and vacancy, make the situation worse, and this issue is becoming critical in cities with high transportation density. For instance, during peak hours, limited roadside spaces are usually occupied by trucks for loading or unloading or by other vehicles for purposes of picking up or dropping off. Some vehicles are required to circle the road network or temporarily double-park while waiting for roadside spaces to become available. Figure 1 illustrates a real-life situation of roadside double-parking, caused by the loading and unloading activities of logistics trucks, company cars and other obstacles. These actions cause unnecessary blocking and create safety issues for other road users, and traffic flow can be affected. Hence, there are increasing numbers of applications for roadside surveillance systems, especially with respect to traffic surveillance related to increasing traffic congestion. However, current roadside surveillance systems have limitations, such as being affected by vehicle speeds [2]. To be useful, a roadside surveillance system requires machine learning and computer vision-based technology to facilitate and enhance the accuracy and effectiveness of detection and recognition of vehicles and objects [3]. With an increasing number of vehicles in roadside situations which may cause severe traffic congestion, a suitable and well-functioning system is needed, utilizing ICT and IoT technologies. This would enable surveillance of roadside loading and unloading bays, to prevent traffic congestion and facilitate traffic and fleet planning and management by implementing smart mobility, hence achieving a highly efficient road network in a smart city. Furthermore, smart transportation and smart parking can be established under the ontology of smart mobility. 

In an attempt to solve these problems, this paper presents an IoT-based system for the surveillance of roadside loading and unloading bays, namely, a computer vision-based roadside occupation surveillance system (CVROSS). By equipping a set of wireless, high-definition vision devices with machine learning and solar power in a vision-based network, real-time roadside traffic images—including recognition of loading or unloading activities—can be observed and captured automatically, to enhance the transparency of roadside occupancy and vacancy. Decision support models for parking-gap calculations and parking-space assignment are formulated, to eliminate the occurrence of double-parking, while fuzzy logic is applied to address the fuzziness in vehicle parking reservations and estimation of the time of stay for vehicles. As a result, the proposed system can evaluate average space utilization, loading and unloading activities and average waiting times for parking. Property management companies and drivers can derive benefits relating to time-saving and smoother traffic flow in busy built-up areas. To validate the proposed system’s performance and feasibility, a case study was conducted in the Kwun Tong District of Hong Kong (one of the busiest districts for roadside loading and unloading activities in Hong Kong). This enabled the formulation of proactive strategies, improving not only efficiency in transportation and traffic flow but also roadside safety for the public. A paired sample t-test was applied to evaluate the hypothesis regarding the difference between estimated parking gaps (generated from the CVROSS) and actual parking gaps, so that the performance of the CVROSS could be validated. In addition, satisfaction and system performance were evaluated by conducting a survey of drivers and property management company representatives who use, and are responsible for managing, roadside areas.

This paper is organized as follows: Section 2 contains a literature review related to this study. In Section 3, the architecture of the CVROSS is presented, demonstrating how it can be applied in Hong Kong, known as one of the busiest cities for road usage. Section 4 presents a case study, implementing the CVROSS to validate its feasibility and performance. Section 5 introduces the results and discusses the findings, together with their implications. Finally, conclusions are drawn in Section 6.

## 2. Literature Review

In this section, an overview of roadside surveillance systems is given. The methods and emerging IoT technologies used in roadside surveillance systems are then discussed, to define the research gap addressed by this paper.

### 2.1. Overview of Roadside Surveillance Systems

With the advancement of technology, roadside surveillance systems have developed significantly in terms of object detection, tracking, classification and behaviour analysis, thus improving accuracy and reliability. Surveillance refers to the processes of focusing systematic and routine attention on certain human behaviours for influencing, managing, protecting or directing purposes [4]. This may entail observation from a distance by means of electronic equipment, such as closed-circuit television cameras (CCTV) or interception of electronically transmitted information, such as internet traffic or phone calls. Surveillance is frequently utilized by governments for intelligence gathering, crime prevention and investigation, or for the protection of a process, person, group or object. For example, traffic surveillance by video cameras is common, using cameras installed in many different locations, e.g., mounted along the highways to monitor real-time traffic situations, thus controlling traffic flow and facilitating traffic management by governments. According to Valera and Velastin [5], roadside surveillance systems are utilized to observe the roadside activities and behaviours of vehicles and road users, and they include moving object detection, recognition, classification, tracking, behavioural analysis and retrieval. This enables real-time monitoring of persistent and transient objects within a specific environment. By applying advanced information and communication technologies, roadside surveillance systems are able to solve transport problems such as road hazards and traffic congestion and, ultimately, they can help to achieve transport efficiency [6]. Smooth traffic conditions are crucial to road users and the public, and traffic congestion can lead to a range of negative effects. According to Robinson [7], traffic congestion is very costly and has an obvious negative effect on productivity, fuel consumption, environmental quality and quality of human life. The effects of traffic congestion include, but are not limited to, delays in journeys, increasing air pollution and carbon dioxide emissions, blocking of emergency vehicles and spillover effects from congested roads to other roads. For instance, traffic congestion may delay the schedules of truck drivers’ loading and unloading activities, thus affecting the management of logistics companies. Some vehicles even circle the road network to wait for available parking spaces. This wastes fuel and energy. Therefore, the ultimate objective of roadside surveillance systems is to facilitate traffic and fleet management, since traffic congestion, accidents and low traffic efficiency result in a waste of resources [8].

### 2.2. Methods of Roadside Surveillance Systems

Video surveillance has drawn the attention of scientists and engineers to active application-oriented research on computer vision, artificial intelligence and image processing. This is a contributing factor in the increasingly widespread deployment of intelligent video-based surveillance systems [9]. One of the most significant applications of intelligent video-based surveillance systems, in which a growing interest has emerged in recent years, is roadside traffic surveillance for dealing with problems of increasing traffic congestion. Current techniques include vehicle detection and tracking to measure traffic parameters and car park management. Traffic conditions are of crucial importance for road users and society generally. Thus, various applications of roadside surveillance systems aim at utilizing image processing methods to obtain better traffic conditions and maintain a high level of road safety, thus achieving a more efficient traffic network [10]. The development of roadside surveillance systems for the measurement of traffic parameters has been a focus of research in recent years. Data on the number of vehicles and their type, speed and flow, are collected by roadside surveillance systems using vehicle detection and tracking techniques. This contributes to transportation planning, traffic operation and pavement design. According to Huang [11], roadside cameras have been applied to estimate lane boundaries and vanishing points, hence classifying vehicles by their physical length, to monitor traffic situations on the roads via video image processing techniques. In addition, Coifman et al. [12] designed a real-time computer vision system for vehicle tracking and traffic surveillance by considering the lighting conditions in daylight and twilight and at night. Saran and Sreelekha [13] also utilized a video-based surveillance system to measure traffic parameters. The system used could be divided into three main functions: detection, classification and tracing of vehicles. This also enabled the number of vehicles to be counted. To provide real-time analytical information, computer vision techniques were used to process images. With the traffic information produced (traffic congestion, number of vehicles, classification, etc.), the video-based surveillance system was expected to achieve and maintain efficient traffic management and road safety. By considering traffic conditions, related applications mainly focus on vehicle detection and tracking, processing the data and images using computing techniques. Hence, useful information about traffic parameters can be produced for further analysis, transportation planning, traffic operation and pavement design.

In addition to the measurement of traffic parameters, the number of applications of surveillance systems for facilitating the management of both indoor and outdoor car parks is increasing. In this type of application, systems are mainly used for counting the number of parked vehicles, monitoring changes in parked vehicles over time and identifying available parking spaces [14]. Searching for parking spaces consumes a significant amount of travelling time, regardless of whether it is in a car park or on the roadside. Moreover, there is always a difficulty in maintaining and providing information in real time without using an intelligent system. Therefore, in recent years, many researchers have studied the possibility of using vision-based car park management systems to determine occupancy and vacancy and to provide users with related information. Lin et al. [15] proposed a vision-based parking management system to manage an outdoor car park, using four cameras set up in the lofts of surrounding buildings to record occupancy and vacancy, then processing the data and transferring the information to users. Greyscale images were captured to maintain colour consistency and increase the accuracy of the system. Furthermore, Micheloni et al. [16] utilized a surveillance system for car parks by managing both static and active cameras in a hierarchical framework. The static camera system employed a variable number of static sensors to maintain the trace of all objects simultaneously, while the active camera system used a pan-tilt camera to capture high definition (HD) video of the target, to prevent problems with multi-sensor, multi-target tracking. These surveillance systems applied to car park management are beneficial for counting the number of parked vehicles, monitoring changes in the parked vehicles over time and identifying available parking spaces. They are also likely to be applicable to roadside loading and unloading bays for locating occupancy and vacancy, thus alleviating the problem addressed in this paper.

### 2.3. IoT Technologies for Roadside Surveillance

Following recent advances in technology, roadside surveillance is not only based on cameras but also on other emerging technologies, aimed at achieving various aims and objectives. Researchers have been most interested in the development and applications of radio tomographic imaging (RTI), wireless sensor networks (WSNs) and computer vision for roadside surveillance. Radio tomographic imaging is an emerging technology that localizes and tracks moving physical objects in an area surrounded by simple and inexpensive radios in wireless networks [17]. The concept of RTI originates from radar systems. Using concepts from radar, RTI originally worked by placing small and inexpensive radios around the area of interest. The radios were able to transmit and receive wireless signals, creating a dense network of links passing through the area. If an object moved within the area, it would reflect or absorb the wireless signal and prevent some of the power from reaching its destination. Hence, an image could be formed of where the power was being absorbed, by utilizing the link’s power-loss measurements. Therefore, it could indicate the locations of any detected and tracked objects. More recently, RTI has been proposed for tracking the location of objects via radio waves, without requiring objects to transmit or receive radio signals. This occurs when the position is extracted by inferring which voxels are obstructing a subset of radio links in a dense wireless sensor network [18]. Anderson et al. [19] demonstrated a novel application of RTI with a secure wireless sensor network for roadside surveillance and vehicle identification and tracking, by combining frames of a moving vehicle into a single image using a vehicle class-identification algorithm. However, the technology of RTI is subject to the speed at which it can detect objects moving through the network [2]. Speed is limited by the time it takes to perform a single scan of the network area. Vehicles on roads are not always static. If some vehicles are travelling at high speeds which are out of the system specification range, RTI might not record anything, thus affecting the process of measuring traffic parameters. Thus, RTI is not totally suitable for all-round roadside surveillance; only for object detection and tracking at relatively low speeds.

WSNs use networked microsensor technology, which is one of the most important technologies of the 21st century and a key technology for the future [20]. Wireless sensor networks offer an attractive, low-cost alternative to inductive loops, video and radar, for traffic surveillance on freeways, at intersections and in car parks. According to Cheung et al. [21], these networks typically consist of a set of sensor nodes comprising a sensor, a microprocessor, a radio, a battery and an access point, together with a radio and a more powerful processor. The sensor nodes are utilized to process real-time measurements and transmit useful data to the access point located at the roadside, through direct communication via either line or solar power. This then enables the detection of vehicles and the estimation of various traffic parameters. Cheung and Varaiya [22] utilized a network of small sensor nodes, communicating wirelessly among themselves, to sense the physical world. After the collection of data from the detection events using the sensor nodes, the access point could calculate the number, occupancy and speed of monitored traffic. It is evident, therefore, that WSNs offer an attractive, low-cost alternative to inductive loops, video and radar, for traffic surveillance. However, Tiwari et al. [23] have argued that WSNs are easily affected by their surroundings, e.g., by walls, microwaves or signal attenuation due to large distances. These networks also have a comparatively low speed of communication with the server and are easily disrupted by elements such as Bluetooth. Due to the instability of communication and concerns about interruption, WSNs may not be appropriate for providing road users and the public with accurate traffic data and information.

Furthermore, technological improvement has led to the development of semi-automatic systems (known as computer vision) for creating algorithms by using computers for automatic real-time object and event detection and to aid recognition [24]. According to Huang [25], from an engineering perspective, computer vision aims at building autonomous systems and seeks to automate, with greater efficiency, tasks that are performed by the human visual system. In fact, several monitoring objectives can be supported by the application of computer vision and pattern recognition techniques, such as detection of traffic violations and identification of road users [26]. Beymer et al. [27] presented a feature-based tracking approach for the task of tracking vehicles during periods of traffic congestion. Video cameras were expected to be mounted on poles or other tall structures looking down at the traffic scene. Huang and Yen [28] designed a real-time and colour-based computer vision system for traffic monitoring, by analysing colour image sequences of traffic scenes recorded by mounting a stationary camera on a tall building or a pedestrian crossing bridge near a traffic light. Although computer vision-based surveillance systems have various functionalities, such as vehicle detection, recognition and classification, the systems require relatively long processing times and considerable memory, since converting collected data into useful information can be challenging. In addition, the set-up for camera calibration to facilitate the extraction of the required images is difficult and time-consuming [29]. However, according to Zander et al. [30], machine learning can be applied to enhance the accuracy and efficiency of detection and recognition of an object, using computer vision-based surveillance systems. Thus, systems are able to learn without being explicitly programmed, by exploring the construction of algorithms. This facilitates more efficient calculation and measurement of real-time information on roadside activities, occupancy, vacancy and traffic parameters.

In summary, an effective roadside surveillance system is critical for maintaining road safety, alleviating traffic congestion and facilitating traffic and fleet management. Applications for the measurement of traffic parameters and car park management utilizing computer vision, show an increasing trend. Computer vision technology can be powerful when properly matched with machine learning and big data analysis, outweighing the disadvantage of long processing times. In addition, this can lead to better analysis, with a high level of accuracy and efficiency [31]. Furthermore, improvements in camera calibration should be considered when utilizing computer vision, to maintain the accuracy, efficiency and effectiveness of the systems and to account for various features on different roads, such as the surroundings and the amount of sunlight.

## 3. Design of a Computer Vision-Based Roadside Occupation Surveillance System (CVROSS)

**Figure 2:**
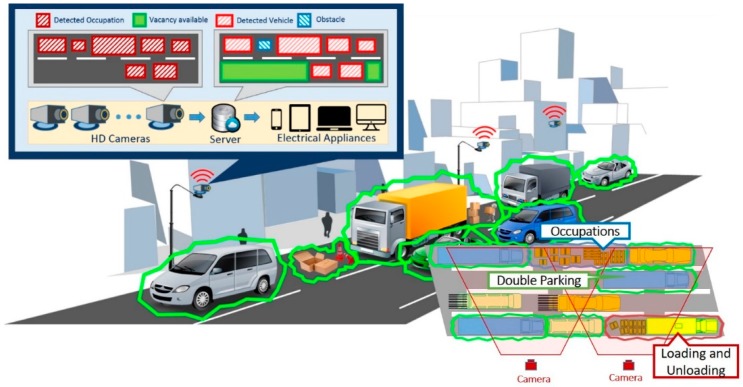
Overview of the CVROSS.

The system design and modelling of the CVROSS are described in this section. Figure 2 shows an illustrative overview of the CVROSS deployed at the roadside. The design of the CVROSS has four major components: (i) roadside surveillance technology using the IoT, (ii) data preprocessing in the CVROSS, (iii) decision support for roadside parking and (iv) evaluation of the proposed system. It aims at reducing issues of double-parking in urban transport systems and improving the visibility of roadside situations. The transparency of roadside occupancy and vacancy can be further enhanced.

### 3.1. Roadside Surveillance Technology Using the IoT

To collect data effectively and efficiently, an IoT-based roadside surveillance system (CVROSS) has been designed and developed to tackle the problem of loading and unloading bays. As shown in Figure 2, the CVROSS was equipped with a set of solar power-enabled wireless HD vision devices, which enable the system to capture images from the roadside. To reduce electrical costs and avoid the risks of relying heavily on external power or solar energy, the devices connect wirelessly to a cloud platform, allowing continuous data transfer to the CVROSS and real-time monitoring of occupancy and vacancy data, retaining up-to-date roadside information for 24 h a day, seven days a week. By using application program interfaces (APIs), the collected data can be examined at the preprocessing stage by the proposed decision support model; thus, the roadside traffic information can be observed in real time. Via a vision module and machine learning, users are provided with hundreds of functions for acquiring images from a multitude of vision devices, for further processing by locating features, identifying objects and measuring parts. In addition, machines can learn from empirical data, making predictions about future data. HD vision devices are expected to be used, to provide the best compromise between maximum observation accuracy and minimum overlapping field of view, to generate the best viewpoint. Image data are then processed by denoising and image tuning, leading to target object detection, recognition, identification, classification and calculation of available parking spaces. As a result, useful and easily accessible traffic information on real-time roadside occupancy and vacancy can be provided to road users. Furthermore, with the aid of machine learning techniques applied in a time-domain dynamic system, both the accuracy and the efficiency of the system are enhanced. Various reports can be generated for road users, logistics companies and the public, for decision-making via big data analysis.

**Figure 3:**
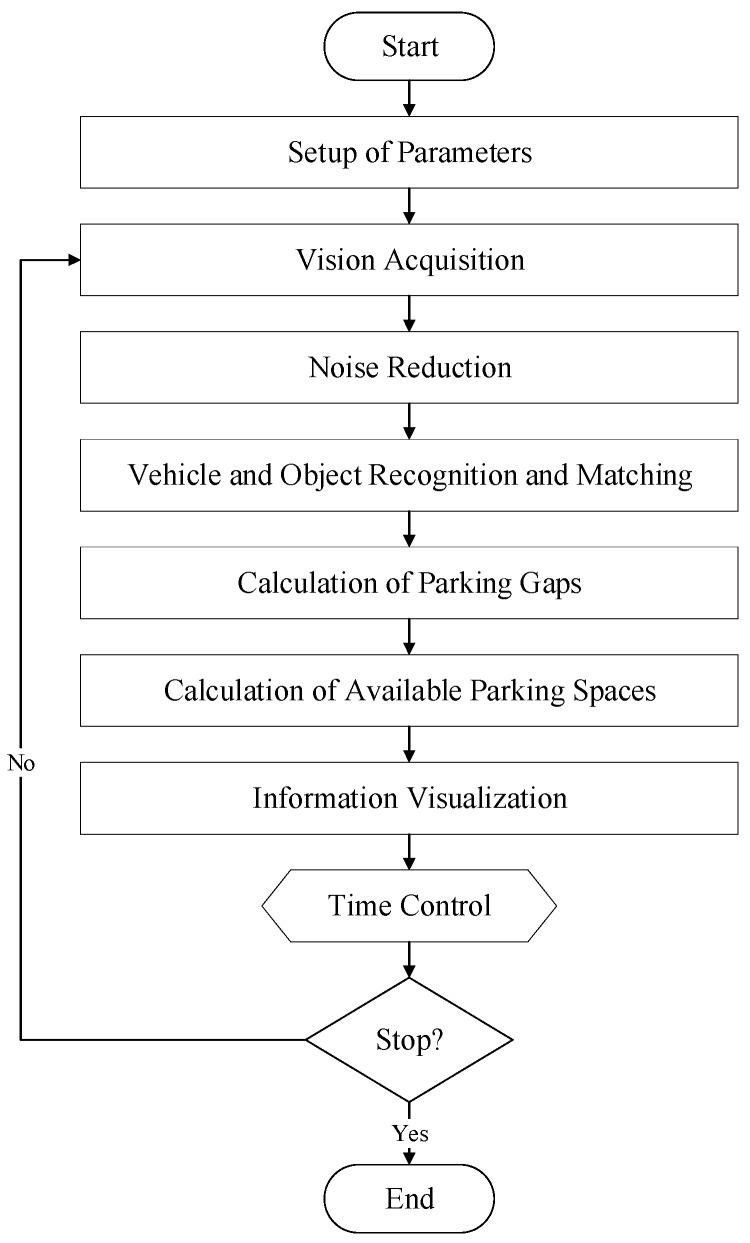
Process flow of the CVROSS.

After installation of the wireless HD vision devices connected to the CVROSS, cloud-based servers can be used with IoT communication protocols. Data analytics and computation modules function as the back-end cloud server, and the results are retrieved and displayed in front-end applications for end users. The process flow of the proposed system is illustrated in Figure 3. Transparency of roadside activities and information can be enhanced and reports can be produced at the end of every timed loop, after image processing.

### 3.2. Data Preprocessing in the CVROSS

Before running the CVROSS, parameters must be set up, including types of vehicles, vehicle parking space regulations and minimum width of traffic lanes. These correspond to the regulations issued by the Hong Kong Special Administrative Region Planning Department [32]. Therefore, the system can compare the captured images with templates in the database, in order to process images and data more accurately in the later stages. To facilitate the calculation of parking gaps and available parking spaces, differences in the dimensions of all items caused by non-identical distances from the vision device are ignored in the computation process. In other words, it is assumed that each of the items presented in a case has the same dimensions in millimetres or pixels, regardless of its position (in terms of distance) in relation to the vision device. In the computation process, the preliminary parameters include:

- 640 × 480 pixels for the entire coverage of the vision device
- 11 m of regulated parking space per truck
- 7 m of regulated parking space per cargo van
- 5 m of regulated parking space per private car
- 6.75 m for the minimum width of traffic lanes
- templates of all possible vehicles and objects
- a confidence score, which indicates the confidence of the disparity for each pixel for each template (image scores return values between 0 and 1000, where 1000 indicates the highest confidence).

After the parameters are input into the CVROSS, the program starts the first stage of the timed loop, i.e., vision acquisition. The wireless HD vision devices connected to the CVROSS can then capture images from the roadside continually and automatically. Afterwards, the collected data are used in: (i) noise reduction and (ii) vehicle and object recognition and matching.

#### 3.2.1. Noise Reduction

**Figure 4:**
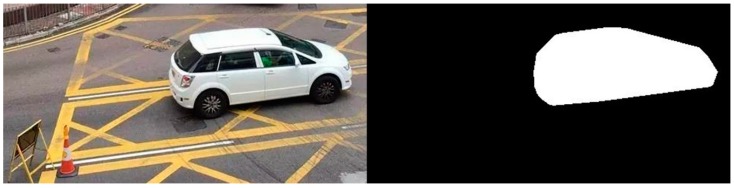
An example of noise reduction.

One of the most important stages in the entire system flow is noise reduction. This is a process of removing noise from an image, as the noise might degrade both the visual quality and the effectiveness of subsequent processing tasks [33]. In this case (according to the simulation model), on the roadside and in traffic lanes, there are different objects and signals, such as traffic indicators and instructions in traffic lanes. However, these are likely to be unrelated to vehicle and object recognition and matching, therefore they may negatively affect matching results and the effectiveness of the subsequent calculation of available parking spaces. Furthermore, even similar vehicles, such as two private cars in this case, may be the same model but different in colour. Therefore, noise reduction can ensure that unrelated objects, indicators and signals are removed before further processing of the images. This also prevents problems with colour classification. In Figure 4, an example of noise reduction is illustrated. Before noise reduction, the image obtained from vision acquisition was full of obstacles, such as a road sign, a traffic cone and yellow box markings. All these were a hindrance to vehicle and object recognition and matching. Noise reduction was achieved using an “image mask” to exclude irrelevant regions of the image, “colour plane extraction” to convert the colour image to a binary image in only black and white, and “basic morphology” to modify the shape of binary objects in the image, as well as to adjust the brightness. After noise reduction, the indicator, traffic cone and yellow box markings had been removed (blacked out) and only the private car remained on the screen with its shape shown in white.

#### 3.2.2. Vehicle and Object Recognition and Matching

Two common matching methods are provided by the CVROSS: pattern matching and geometric matching. If all the items that need to be detected and matched share the same features, pattern matching is the best method, as it will compare all the features and colours of an item from the template and the captured image. However, not all vehicles and objects are the same. For example, some owners may paint the roof or body of a vehicle. Therefore, not all objects have the same patterns or the same colours. This may negatively impact on the effectiveness of vehicle and object recognition and matching. As a result, together with noise reduction (converting the captured image to a binary image in only black and white), geometric matching seems more suitable for use in the CVROSS to detect, recognize and match different types of vehicles and objects based on their shapes, lengths and other significant features, as well as to determine the image score values mentioned. It can prevent failure of recognition and matching of an item due to different patterns and colours. In this case, when the image is acquired properly and noise has been reduced, the process of recognition and matching can then be carried out. Vehicle and object recognition and matching are based on templates inserted during the set-up process for the system parameters. When an object appears, or a vehicle passes by or parks inside the angle of view of the HD vision devices, the devices will capture images and compare them automatically with the templates in the database. Thus, vehicles and objects can be assigned to a category after recognition.

### 3.3. Decision Support in Roadside Parking

#### 3.3.1. Evaluation of Parking Gaps

**Figure 5:**
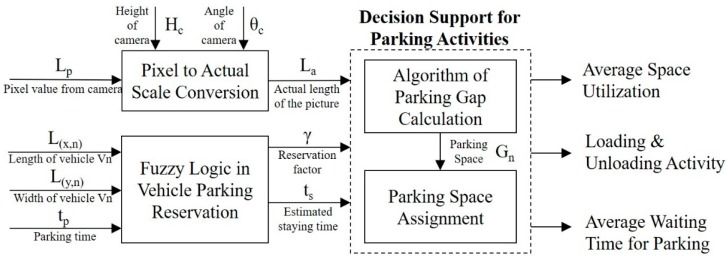
Overview of the computations in the CVROSS.

**Figure 6:**
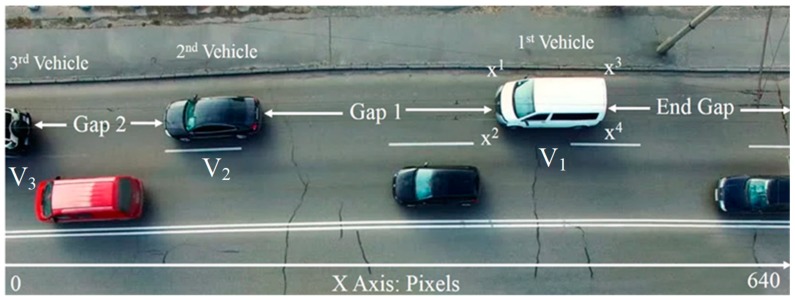
Illustration of calculation of parking gaps.

In the following sections, parking-gap calculations, parking-space evaluation and decision support in parking are considered and evaluated, as shown in Figure 5. The block diagram shows that the entire computation involves three components: (i) conversion between pixel values and actual scale for road traffic, (ii) fuzzy logic for vehicle parking reservation and (iii) decision support for parking activities. After vehicles and objects have been recognized and matched, the CVROSS calculates parking gaps for each individual traffic lane. First, the conversion between the pixel value collected from the image and the actual scale should be implemented, via experimental studies. The actual scale of the road traffic is affected by the height of the camera (H_c_) from ground level and the viewing angle of the camera (θ_c_). The conversion ratio is essential for estimating the actual number of parking spaces and is utilized in the following analysis. As indicated in Figure 6, in the first traffic lane, shown at the top of the image, there are three vehicles, and each of them has four corner points, i.e., {(*x_1_*, *y_1_*), (*x_2_*, *y_2_*), (*x_3_*, *y_3_*), (*x_4_*, *y_4_*)} ⊆ {X_1_, Y_1_} for the first vehicle V_1_. In general, the four corner points of vehicle V*_n_* can be presented in the form {(*x_4n-3_*, *y_4n-3_*), (*x_4n-2_*, *y_4n-2_*), (*x_4n-1_*, *y_4n-1_*), (*x_4n_*, *y_4n_*)} ⊆ {X*_n_*, Y*_n_*}, where *n* is the number of vehicles captured in the image.

**Figure 7:**
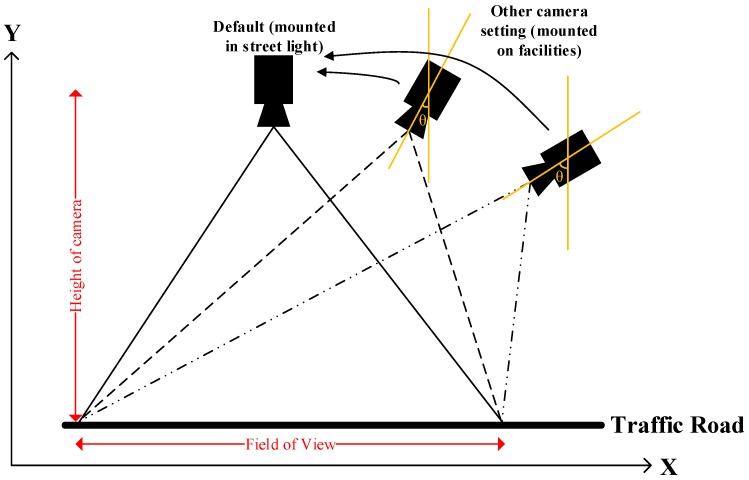
Illustration of various camera positions.

In this study, it is supposed that the cameras are mounted on street lights and nearby facilities, so that the heights and viewing angles of the cameras may be different. Thus, adjustment of the images taken by cameras that are not mounted on street lights is needed, in order to standardize the image for conversion. Referring to the government’s street-light design [34] and other work [35,36], a conversion ratio mapping can be established to deal with various predefined heights and angles of the cameras, in order to standardize their field of view (FOV). Figure 7 illustrates the scenario of mounting cameras at different heights with different viewing angles. In the default setting, the cameras that are mounted on the street lights are set vertically, to cover a particular FOV. For other camera settings with different heights and viewing angles, the image and the FOV are then adjusted back to the default setting. To achieve the above goal, a conversion mapping profile is established via experimental studies for several predefined configurations, and thus the ratio $L_{a}/L_{p}$ is defined for the conversion between pixel values and actual scales for road traffic. Consequently, the conversion ratio can be applied for calculating the actual length and width of vehicles. 

After the four corner points of each vehicle have been retrieved, the CVROSS computes the maximum and minimum values of x and y, i.e., L_(*x*,*n*)_ and L_(*y*,*n*)_, to represent the actual length and width of vehicle V*_n_* respectively, according to Equations (1) and (2). Using the above information, the size of the vehicle captured by the camera is known, and the spaces for vehicle parking reservation are then computed using fuzzy logic:

(1) $$L_{\left(x,n\right)}=[\max\left(X_{n}\right)-\min\left(X_{n}\right)]\cdot \frac{L_{a}}{L_{p}}$$

(2) $$L_{\left(y,n\right)}=\left[\max\left(Y_{n}\right)-\min\left(Y_{n}\right)\right]\cdot \frac{L_{a}}{L_{p}}$$

In fuzzy logic, there are three processes: fuzzification, the inference engine and defuzzification. These evaluate the reservation factor γ ∈ [0, 1] and the estimated time of stay *t_s_* from three inputs, i.e., parking time *t_p_*, L_(*x*,*n*)_ and L_(*y*,*n*)_. In fuzzification, the input and output parameters are fuzzified with a set of defined fuzzy classes, such as “small”, “medium” and “high”, and the degree of belongingness *μ*, all taking values between zero and one. For example, the parking time *t_p_* is fuzzified with its corresponding membership functions to show the degree of belongingness, as in Equation (3) where *x_i_* represents all elements in *t_p_*, *μ_A_*(*x_i_*) is the membership function of fuzzy class A in *x_i_* and *n* is the total number of elements *x*. In the inference engine, the set of rules R = {R_1_, R_2_, R_3_,…, R*_m_*} is used to evaluate the aggregated outputs from the input parameters, where *m* is the total number of rules collected from interviewing the domain experts. The mechanism of the inference engine is referred to as Mamdani’s method [37], where the consequences of the rules are expressed by fuzzy sets rather than linear mathematical expressions. Equation (4) shows the inference process for obtaining the aggregated outputs. The “OR” operator is applied for combining all the membership function values, resulting in a bounded area in the output membership functions. In defuzzification, the outputs in fuzzy sets are then converted back to crisp values *x′* using the centroid method, which measures the centre of gravity of the bounded area, as shown in Equation (5). In the application, when the region of the vehicle is recognized in the image, the parking time, reservation factor and estimated time of stay for the vehicle can be measured, to truly reflect the occupied spaces for vehicle parking. The membership functions used in fuzzy logic are predefined intuitively by interviewing domain experts and industrialists, as shown in Table 1:

(3) $$t_{p}=\sum_{i=1}^{n}\frac{\mu_{A}\left(x_{i}\right)}{x_{i}}$$

(4) $$\mu_{B}\left(Y_{i}\right)=\max\left\{\min_{i}\left[\mu_{A_{1}}\left(x_{1}\right),\;\mu_{A_{2}}\left(x_{2}\right),\;\ldots ,\;\mu_{A_{j}}\left(x_{j}\right)\right]\right\}$$

(5) $$x^{\prime }=\frac{\int \mu_{A}\left(x\right)\cdot xdx}{\int \mu_{A}\left(x\right)dx}$$

**Figure 8:**
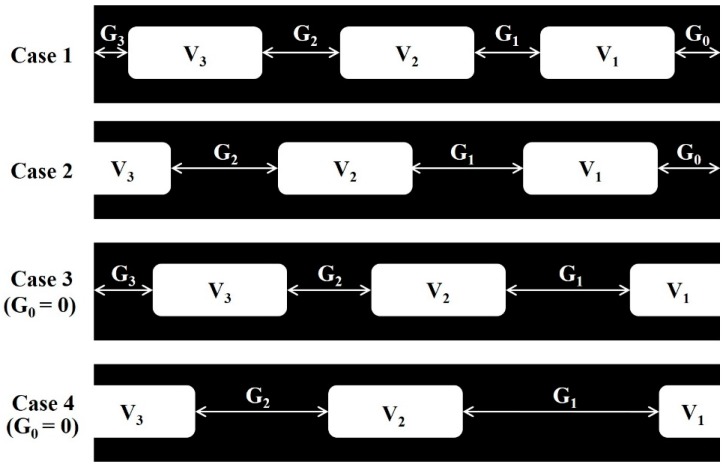
Four possible cases for capturing images.

To calculate the parking gap between vehicles, four situations should be taken into consideration, as shown in Figure 8. To prevent errors of unlimited value, the largest pixel value for length (the 640th pixel rather than the first pixel), is utilized to compute the first gap, namely, the end gap (G_0_). Hence, in accordance with Equation (6), the end gap (G_0_) can be computed by subtracting the maximum *x*-value of the first vehicle V_1_ from the maximum pixel value for length, i.e., 640 pixels. For cases 1 and 2 in Figure 8, the calculation of the end gap is performed via Equation (6), and the end gap is the partial parking gap between two vehicles, such that the information from the right camera should be considered to measure the whole parking gap between the two vehicles (as for case 1 or 3). For cases 3 and 4 in Figure 8, since the maximum *x*-value of the first vehicle V_1_ is equal to the maximum pixel value, this implies that the end gap is equal to zero and the length of the first vehicle captured by the camera does not represent the actual length of the vehicle. The information from the right camera should be collected and combined with the partial length of V_1_ to confirm the actual length of V_1_, whilst the situation of the left camera should be similar to case 2 or 4. In the algorithm, the parking gap G*_n_* between vehicles *n* and *n*+1 can be generalized for one specific traffic lane, as shown in Equation (7). The parking gap (in pixel values) can be obtained and can be converted back to the actual scale using the pixel-to-actual-scale conversion ratio. When considering cases 1 and 3, X*_n+_*_1_ cannot be observed for computing G*_n_*, so that max(X*_n+_*_1_) is defined to be zero, which defines the edge of the captured image. All the above situations were considered in the proposed system, and parking-space assignment was then conducted for three types of vehicles: a private car, a cargo van and a truck, with regulatory parking spaces of 5 m, 7 m and 11 m respectively [35].

(6) $$G_{0}={\mathrm{Pixel}}_{\max}-\max\left(X_{1}\right)$$

(7) $$G_{n}={\mathrm{Pixel}}_{\max}-\max\left(X_{n+1}\right)-\sum_{i=0}^{n-1}G_{i}-\sum_{j=1}^{n}[L_{\left(x,j\right)}\cdot \gamma_{j}\cdot \frac{L_{p}}{L_{a}}],\;\mathrm{where}\;n\neq 0$$

#### 3.3.2. Parking Spaces and Decision Support Functionalities

Calculation of parking gaps is useful for computing the available parking spaces. After gathering data on parking gaps in traffic lanes, the CVROSS can carry out further processing by dividing the length of each gap by the lengths of different types of vehicles *ω* (input as one of the parameters described in Section 3.3.1), via Equation (8) for a truck, van and private car. If the length is less than the length of a private car, the objects are classified as “others”, e.g., obstacles in the road. Therefore, the information on available parking spaces (S_truck_, S_van_ and S_private car_) for the three types of vehicles in each individual traffic lane, or even for the whole road, is produced, to inform road users about real-time roadside occupancy and vacancy. Consequently, the proposed system can provide three decision support functionalities: evaluation of average space utilization, measurement of loading and unloading activity and average waiting time for parking. Considering that there are p cameras in the whole traffic lane, the average space utilization (U) is calculated by dividing the total available parking gaps by the maximum length of the image (in pixels), as shown in Equation (9), where G*_ij_* represents the available parking gap *i* determined by camera *j* and Pixel_(*j*,max)_ represents the maximum pixel value of camera j. If the traffic space is occupied by trucks and cargo vans instead of private cars, these are regarded as engaging in loading and unloading activities. The indication of performing loading and unloading activities (LUA) is calculated by dividing the sum of the lengths of the vehicles, for L_(*x*,*n*)_ ≥ 5, by the actual total length of the traffic lane (L_traffic_) under surveillance by camera *p*, as in Equation (10). The indication of loading and unloading activities is assumed to be updated hourly in the proposed system, to conveniently track the traffic situation. For average waiting times for parking, the proposed system will determine the waiting time for the entire traffic lane when any available parking space G*_n_* is less than the required parking space for a private car (representing the smallest parking space for the three types of vehicles). Therefore, users can make an appropriate decision according to the above three indicators:

(8) $$S_{k}\;=\frac{1}{\omega }\cdot [G_{n}\cdot \frac{L_{a}}{L_{p}}],\;\mathrm{where}\;k=\mathrm{truck},\;\mathrm{van}\;\mathrm{or}\;\mathrm{private}\;\mathrm{car};\;\omega =5,\;7\;\mathrm{or}\;11$$

(9) $$U=\frac{\sum_{j=1}^{p}\sum_{1=1}^{n}G_{ij}}{\sum_{j=1}^{p}j\cdot {\mathrm{Pixel}}_{\left(j,\max\right)}}$$

(10) $$\mathrm{LUA}=\frac{\sum_{j=1}^{p}\sum_{i=1}^{n}L_{\left(x,i\right)}^{j}}{L_{\mathrm{traffic}}},\mathrm{for}\;L_{\left(x,i\right)}^{j}\geq 5$$

In addition, the CVROSS was run with a timestamp control. Frequently updating real-time information allows road users to obtain useful information about real-time roadside occupancy and vacancy. Thus, road users can make good use of the information to make real-time decisions, such as parking their vehicles on the road or finding other roads. In addition, running the program with a time delay can prevent overrunning and overloading of the server, thus maintaining a high level of stability and accuracy of calculation. 

### 3.4. Evaluation of the Proposed System

**Figure 9:**
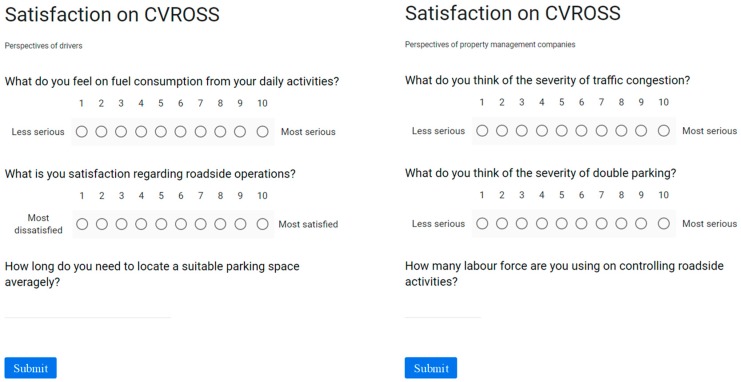
Sample of survey questions for evaluating system performance.

The evaluation of the proposed CVROSS system is twofold: (i) validation of parking-gap estimations and (ii) system performance from the perspectives of drivers and property management companies. The parking-gap estimation is validated using a paired sample t-test for examining the difference between two sets of 50 sample data points, (i.e., estimated and actual parking gaps). This is aimed at investigating the appropriateness of parking-gap estimations in the CVROSS. On the other hand, the satisfaction and the system performance are evaluated by interviewing drivers and property management company representatives, using a survey. Figure 9 shows sample questions used to obtain feedback. The survey was conducted in two timeframes: (i) before implementing the CVROSS and (ii) three months after implementing the CVROSS. As a result, a comparative summary before and after implementing CVROSS was produced, for further statistical analysis.

## 4. Case Study

To validate the feasibility and performance of the proposed system, a case study was conducted, implementing the CVROSS in Kwun Tong District, Hong Kong. Due to the seriousness and urgency of the problem of traffic congestion in Hong Kong, particularly in Kwun Tong District, an IoT-based system for surveillance of roadside loading and unloading bays is much needed. The entire implementation was divided into three phases: (i) site selection, (ii) deployment of the CVROSS and (iii) establishment of web-based user interfaces. The project commenced with data collection mainly from selected site visits focusing on Kwun Tong District. Having obtained a better understanding of traffic situations and occupation, a simulation model was built based on traffic features and real cases in Kwun Tong District. Subsequently, a solution with the CVROSS system architecture was deployed to tackle the issue with the help of a web application. Thus, implementing the CVROSS involved the application of computer vision, cloud computing, big data analysis and reusable energy (solar power), to detect, recognize and match vehicles and objects, hence providing road users with comprehensive and real-time information, after image processing. The information was also visualized using a front-end web interface to enhance understandability.

### 4.1. Site Selection

**Figure 10:**
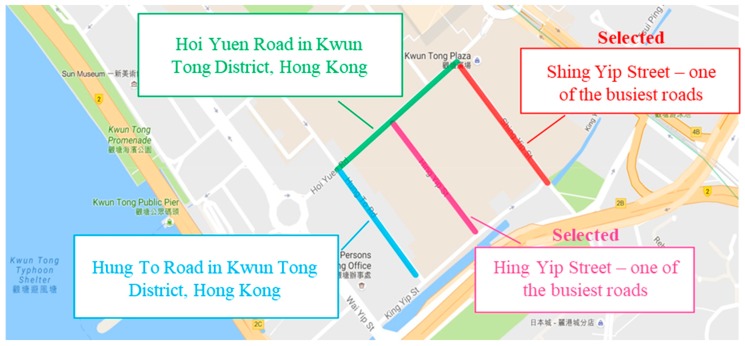
Selected traffic lanes for site visits.

In this phase, data collection was mainly focused on site visits in Kwun Tong District, Hong Kong, in order to gather traffic information from real situations for further analysis. The detailed site visits were carried out on Shing Yip Street and Hing Yip Street, next to Hung To Road and a branch of Hoi Yuen Road in Kwun Tong District, which are the busiest roads in the area (as shown in Figure 10). The data collected included traffic facilities and information on the surroundings of the selected roads, such as the number of lamp posts, traffic lights and traffic lanes, together with distances and the lengths and widths of the roads. Data on traffic situations in the area considered were collected through observation, to obtain a better understanding of the real state of occupation and traffic congestion.

As a major industrial area, Kwun Tong District sees a large number of loading and unloading activities every day. Firstly, vehicles temporarily double-parked for loading or unloading or waiting for roadside spaces to become available, are common in the area of interest. Because a large number of trucks usually double-park on Hing Yip Street, serious traffic congestion can occur. Secondly, as parking spaces are scarce in Kwun Tong District, some nearby companies might occupy the roadside with objects such as traffic cones and boards, to preserve parking spaces. This situation hinders other road users from using the road. Therefore, in addition to detection, recognition and classification of various vehicles and objects, the computer vision-based roadside surveillance system needs to provide road users and logistics companies with information about occupancy and vacancy, so that they can optimize fleet schedules based on analytical information via self-regulation. 

External walls of buildings and lamp posts are the only possible positions for installing the HD vision devices for capturing images in the computer vision-based surveillance system. There is some difficulty in installing HD vision devices on the external walls of buildings, particularly on commercial buildings, without permission. It is believed that most property owners are likely to refuse to install the HD vision devices due to a lack of benefits and effects on the appearance of their buildings. Furthermore, various buildings may have different features at different heights, and this may lead to difficulties in unifying standards, such as the height of all HD vision devices, thus negatively affecting vision and possibly creating some overlaps. Therefore, lamp posts, managed by the Highways Department of the Hong Kong Government, are recommended as the best places to install the HD vision devices along roads and streets. In the areas considered, lamp posts have a mounting height of 10 m, set by the Highways Department [32]. The Highways Department is responsible for preventive and corrective maintenance of lamp posts. This is beneficial for the installation and operation of the vision devices and computer vision-based surveillance system, as breakdowns and errors can be resolved promptly to maintain a high level of stability in the system, compared with installations on the external walls of buildings.

### 4.2. Deployment of the CVROSS

**Figure 11:**
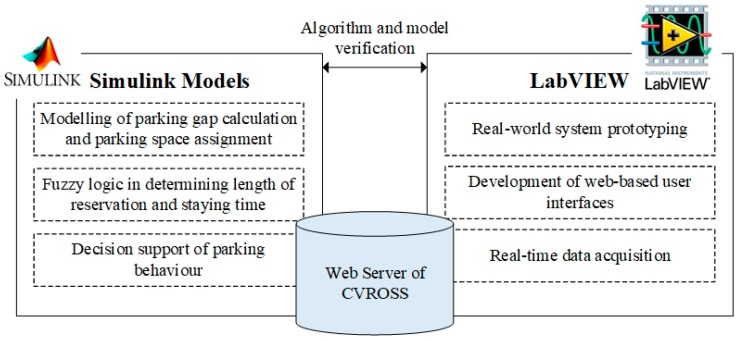
System deployment environment of the CVROSS.

In the design of the CVROSS, the deployment of the proposed system consists of four major components: (i) noise reduction, (ii) vehicle recognition, (iii) calculation of parking gaps and (iv) calculation of available parking spaces. The proposed system was deployed using Simulink and LabVIEW for algorithm modelling and real-world prototyping respectively, as shown in Figure 11. The models and algorithms for parking-gap calculation and parking-gap assignment and the fuzzy logic for vehicle parking reservation, were developed in the Simulink environment, while the user interface and the system prototyping and data acquisition elements, were controlled and constructed in the LabVIEW environment. 

#### 4.2.1. Noise Reduction

**Figure 12:**
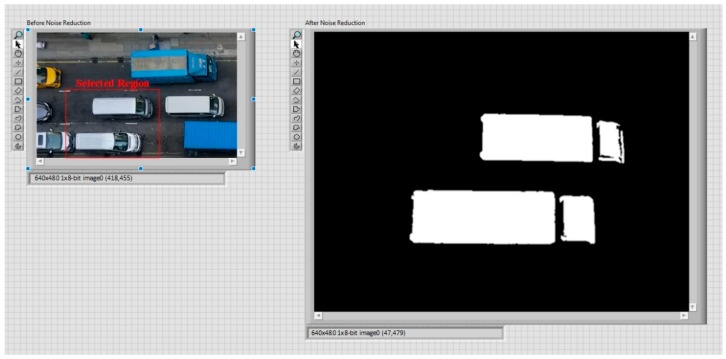
Image differences before and after noise reduction.

Firstly, the set-up of the parameters for real-life implementation was required, especially the size of vehicles and traffic cones and their dimensions in pixels, according to Section 3.2. Then, data collection commenced via vision acquisition and noise reduction. As shown in Figure 12, the original image from vision acquisition included the traffic-lane lines, which are unrelated to vehicle and object recognition and matching. These lines are likely to negatively influence the results of recognition and matching. It was found that, after noise reduction, only the shapes of related vehicles remained. This could facilitate the subsequent processing of the image. As a result, better and more accurate processing could be achieved, to enhance the effectiveness of the designed system. However, different roads with their own features and characteristics may require different techniques for noise reduction, to remove unrelated signals from an image. This may represent a time-consuming modification when the system is applied to different roads in Hong Kong.

#### 4.2.2. Vehicle Recognition

**Figure 13:**
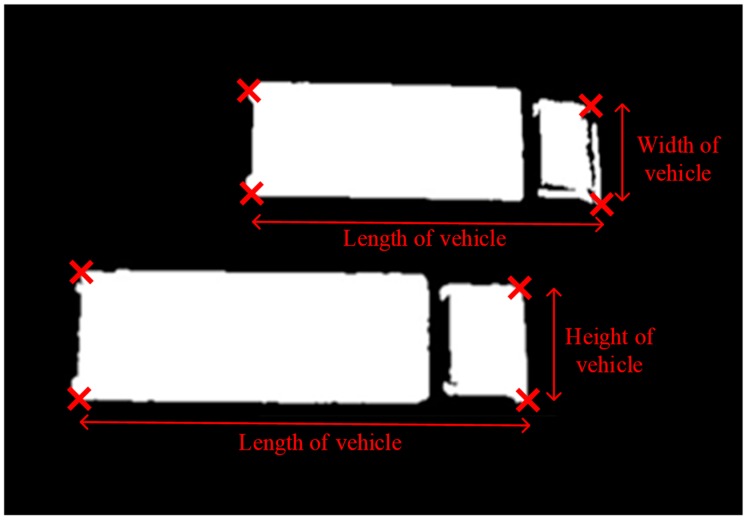
An illustration of geometric matching by the CVROSS.

To determine the dimensions of specific vehicles, the technique of geometric matching was used to recognize and match two trucks with different appearances. It was found that the CVROSS was able to detect, recognize and match vehicles from all traffic lanes on the road and those in an individual traffic lane. General traffic conditions could be interpreted using the information from the matching results for all traffic lanes on the road, for example, for the issue of vehicles remaining double-parked. On the other hand, information from an individual traffic lane was capable of illustrating situations in a particular traffic lane, to determine the level of traffic congestion and identify loading and unloading activities. Figure 13 illustrates the use of geometric matching in vehicle recognition. The coordinates of vertices are located for measuring the corresponding length and width of the vehicles via a geometric technique.

#### 4.2.3. Calculation of Parking Gaps

**Figure 14:**
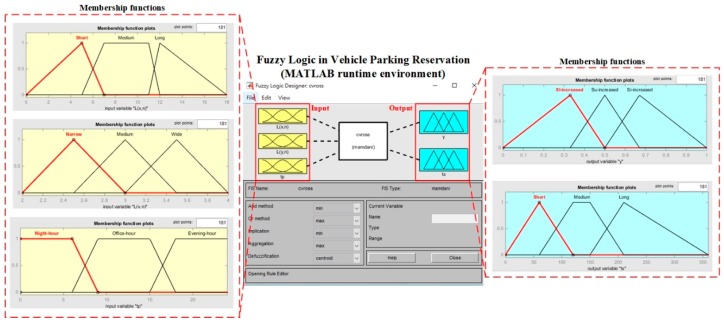
Fuzzy logic in MATLAB runtime compiler.

Using the matching results, the calculation of parking gaps was implemented. The roadside situations (at most six gaps, including the end gap) could be determined by one HD video camera. The total number of gaps was set to a large number *M* for the implementation. By applying the CVROSS, the parking gaps (considering various types of vehicles) could be identified and computed, for cases where *L_p_*, *H_c_* and *θ_c_* are well-defined. Figure 14 shows the fuzzy logic toolbox running in the MATLAB runtime compiler on the server side of the CVROSS. The input and output—with their corresponding membership functions as given in Table 1—were created, and the fuzzification process, Mamdani’s inference and the defuzzification process were defined. Thus, the fuzzy capability of parking reservation can be included in the computation of parking gaps. 

**Figure 15:**
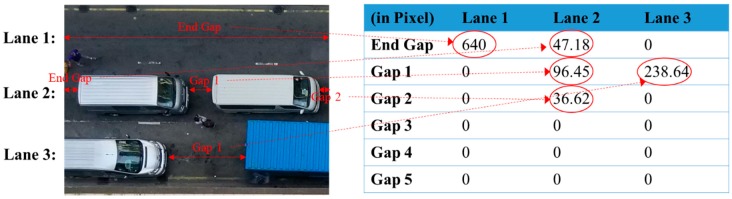
Parking gaps in the three individual traffic lanes.

Figure 15 illustrates the lengths of the parking gaps in three lanes with different numbers of vehicles. When there was no vehicle in lane 1, the end gap was displayed as 640 pixels, which indicated that all the pixels were available, while the other gaps had zero value. From the computations, it was found that the algorithm was able to calculate the parking gaps programmatically, based on the previous matching results. For example, if no vehicles are in the traffic lane, only the end gap is shown. As a result, information on the length of all parking gaps can be produced and used for further processing. However, the number of gaps needed to be set before running the program. Thus, further calculation was required regarding the capacity of a particular road and traffic lane. Then, the possible number of parking gaps that may need to be computed was set.

#### 4.2.4. Calculation of Available Parking Spaces

**Figure 16:**
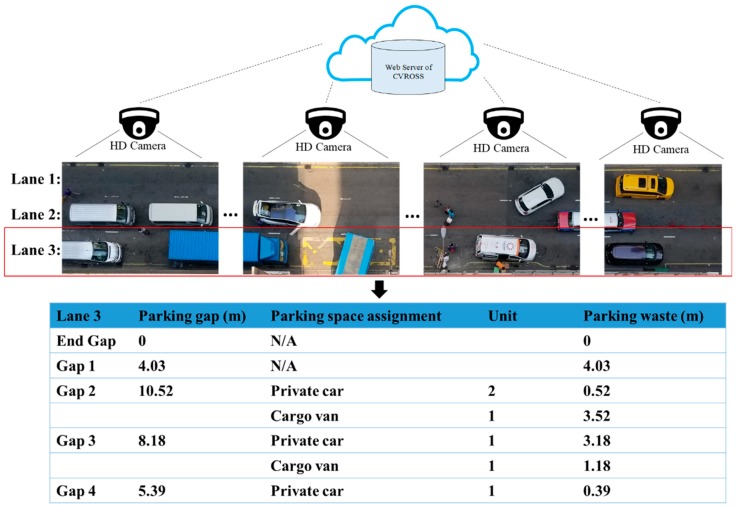
Calculation of available parking spaces in the case of occupation by an object.

The calculation of available parking spaces was then tested by applying the CVROSS, after obtaining the data on parking gaps. Based on the proposed algorithm, the length of each parking gap (in pixels) was divided by the length of each type of vehicle and the constant reserved for parking that particular vehicle. The number of vehicles available for parking in the individual traffic lane was computed by the algorithm. If the number of private cars (which are the shortest vehicles in the scenario) was equal to zero, this meant that a particular parking gap was wasted. Furthermore, the CVROSS was able to add up the lengths of all vehicles and available parking spaces, and show these as occupancy and vacancy respectively, as well as showing the wasted spaces in the individual traffic lane, to provide users with information on the general traffic conditions. Therefore, by dividing the length of parking gaps by the length of each type of vehicle, the CVROSS—using the function for calculation of available parking spaces—proved its ability to compute, and provide users with, information about the number of spaces available for parking in each area. Thus, it is useful and helpful to users in making immediate decisions and for self-regulation. For example, if there are no longer any available parking spaces, drivers can decide to park their vehicles on other roads, to prevent traffic congestion occurring. Furthermore, the CVROSS is also applicable to object recognition and matching. Figure 16 shows the aggregation of available parking spaces in specific lanes. The data on parking gaps collected from several cameras are summarized to form the set of aggregated results of parking gaps and used to assign various types of vehicles to the empty gaps. Thus, the CVROSS is still able to compute the parking gaps and available parking spaces to show occupancy and vacancy, as well as the wasted spaces, for the situation where there is occupation by an object in the traffic lane. 

### 4.3. Establishment of Web-Based User Interface

**Figure 17:**
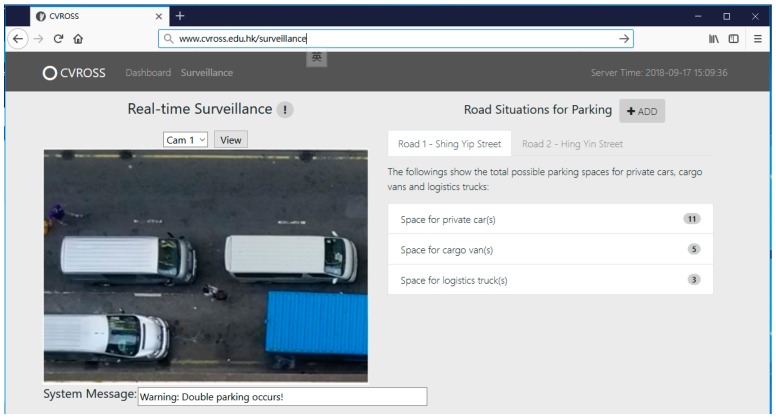
Overview of the interface design for information visualization.

**Figure 18:**
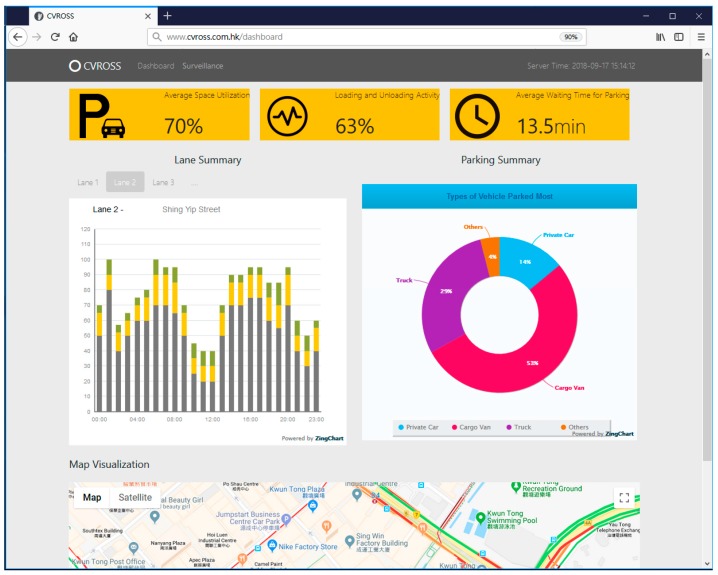
Main dashboard of the CVROSS.

It is crucial to provide users with a good-quality interface for information visualization that is easy to understand and simple to use. The CVROSS is able to offer users various forms of information visualization. The results of vehicles detected and matched, as well as the parking gaps and available parking spaces computed, can be presented graphically. An interface design for information visualization is presented in Figure 17. An image display was utilized to present real-time traffic situations on the road within the system time. This could provide users with a general view of the areas under surveillance. In addition, as shown in Figure 18, on the main dashboard of the CVROSS, various reports about road usage by different types of vehicles can be generated for road users, logistics companies and the public, for better understanding of traffic situations in the areas under surveillance in a particular period.

## 5. Results and Discussion

**Figure 19:**
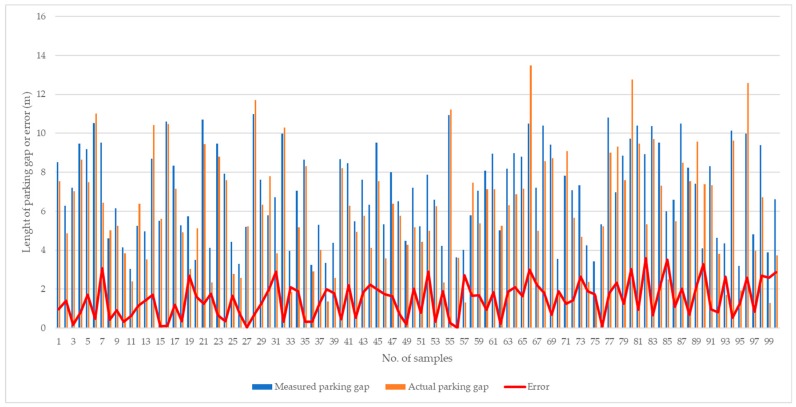
Accuracy comparison for parking-gap measurement.

**Figure 20:**
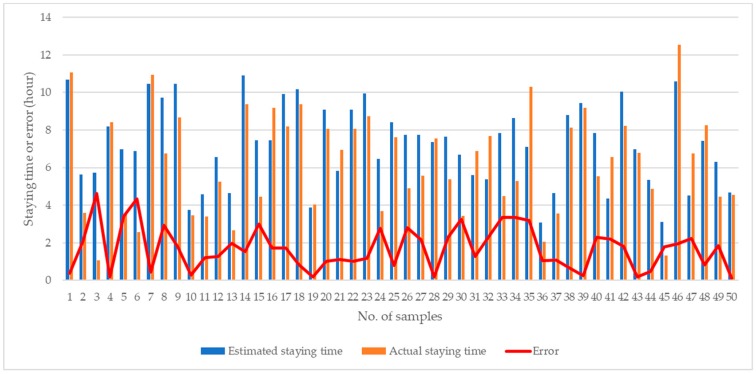
Accuracy comparison for estimation of time of stay.

Following the case study, it was found that it was feasible to implement the proposed system in real-life situations, to provide functionalities for real-time monitoring and decision support for roadside parking activities. On the one hand, property companies can evaluate the severity of double-parking and view the real-time roadside situation from the back office. During the case study, 100 samples of parking gaps were examined for accuracy of parking gap estimation, compared with the actual measured parking gap. The accuracy comparison between estimated and actual parking gaps is shown in Figure 19. A paired sample t-test was applied to examine the difference between estimated and actual parking gaps, and the null hypothesis was to assume that the mean difference was zero. It was found that statistical significance for the mean difference was achieved with a *p*-value of 0.02; thus, the measurement of parking gaps using the CVROSS can be considered feasible. The average and maximum errors for parking gap estimation were 1.47 m and 3.60 m, respectively. Moreover, the accuracy of estimation of the time of stay was also assessed using 50 samples to compare estimated and actual times of stay, as shown in Figure 20. A paired sample t-test was also applied to examine the difference between estimated and actual times of stay, and the null hypothesis was to assume the mean difference was zero. It was found that statistical significance for the mean difference was achieved with a *p*-value of 0.015; thus, the estimation of time of stay can be considered feasible. The average and maximum errors in the estimation of time of stay were 1.70 h and 4.64 h, respectively. On the other hand, truckers and drivers can make use of the proposed system to understand the specific roadside situation. In the next section, the performance of CVROSS is assessed by conducting a comparative analysis—before and after adopting the proposed system. Any advantages and contributions are discussed accordingly. 

### 5.1. Comparative Analysis of the CVROSS

To verify the performance of the proposed system, a comparison of before and after the use of the CVROSS was made, considering three aspects: (i) severity of traffic congestion, (ii) energy savings of vehicles and (iii) driver satisfaction. The results were obtained by interviewing 50 drivers and 10 representatives of property management companies in the selected areas. These were selected because they were frequent users of Shing Yip Street and Hing Yip Street and had considerable management responsibility. Table 2 shows the findings from the interviews with property management company representatives and individual drivers. In summary, the effects obtained by implementing the proposed system appear to be positive. According to property management companies, the severity of traffic congestion on specific roads and incidences of double-parking were reduced by 41.2% and 33% respectively. Moreover, companies can save on the labour force costs of controlling the busy roadside situation, reducing numbers of workers from 10 per shift to six per shift. Monitoring and control of roadside situations can be conducted in the back office, and real-time traffic information can be provided to truckers and drivers via the proposed system. Drivers and truckers commonly agreed that average fuel consumption was reduced, and that the average time to locate suitable parking spaces could be decreased by 51.6%, as they were able to go to specific parking areas after receiving information from the proposed system. In addition, truckers and drivers were generally satisfied with the proposed system, as it could improve the poor situation regarding double-parking and traffic congestion in busy districts.

### 5.2. Timestamp Control of the CVROSS

A while loop (a control flow statement that allows code to be executed repeatedly) can also be utilized in the servers. This allows the program to be run every second or even more frequently, also enabling data, information and reports to be saved every second or more frequently. Although this can provide users with regularly updated information, the server overloads easily, as large quantities of data, information and reports must be processed and saved. Thus, the stability of the system is negatively affected. For example, assuming a month has 30 days, there are 2,592,000 s in a month (for an Excel file generated programmatically utilizing a while loop). This means that there are 2,592,000 records per month, generating approximately 31,536,000 records per year. This could be problematic for big data analysis after a number of years, as more data are generated.

As a result, a timed loop is suggested instead of a while loop. In this case, the timed loop is set with a five-minute delay. Thus, it can still update the real-time traffic information frequently for road users, facilitating the operations of the CVROSS and loading the server smoothly, to prevent overloading and to maintain stability, facilitating the process of big data analysis. In fact, the five-minute delay can be adjusted, based on the real needs of road users, hence providing them with a more user-friendly system.

### 5.3. Significance of the CVROSS

The feasibility and the performance of the CVROSS have been demonstrated by the case study and implementation. The proposed system makes three major contributions to research and society: (i) smart parking for roadside operations, (ii) applied artificial intelligence for roadside parking activities and (iii) an environmentally-friendly business model for property management companies.

#### 5.3.1. Smart Parking for Roadside Operations

In the field of urban development, smart cities are thought to be a future trend and emerging technologies are applied to formulate different forms of decision support and intelligence, to improve efficiency and effectiveness. In the evolution of the smart city, smart mobility is specific to objects (including human beings), transportation and logistics. The ontology of smart parking is developed from smart transportation, which is an active research area. In this paper, smart parking for roadside operations was applied, to eliminate double-parking and enhance roadside occupancy. Via the adoption of IoT technologies, the new topic of smart parking has been explored to address the problem of double-parking at the roadside. Therefore, novel contributions relating to smart parking have been made in this paper.

#### 5.3.2. Applied Artificial Intelligence for Roadside Parking Activities

In the evaluation of parking gaps and available parking spaces, the proposed system makes use of fuzzy logic to classify various types of vehicles in real-life situations, i.e., private cars, cargo vans, trucks and obstacles. Fuzzy logic offers flexibility and intelligence in the algorithm, to generate certain decision support functionalities. Therefore, the proposed system is able to evaluate reservation spaces and the estimated time of stay of the vehicles. This information can be used to estimate average space utilization, loading and unloading activity and average waiting times for parking. Overall, the data collected by IoT technologies and the data on roadside activities are integrated using artificial intelligence techniques, (i.e., fuzzy logic), to create value in industrial applications.

#### 5.3.3. Green Business Model Using the IoT

Considering the findings from implementing the proposed system, it can be concluded that the proposed system has advantages with respect to energy-saving, time efficiency and better roadside occupancy. For example, if there are always many trucks loading and unloading on Monday mornings, some logistics companies can plan to change their schedules in order to load or unload goods at other times, to prevent waiting at times of traffic congestion. Thus, the system is able to facilitate traffic and fleet management by self-regulation. The interface and related information can be further amended and transferred to a mobile application to enhance the transparency of roadside activities. Via self-regulation by road users and logistics companies (taking advantage of information and communication technologies), the system relieves traffic congestion, achieves an efficient road network and facilitates the development and management of a reliable and intelligent transport system. This work is not only beneficial to property management companies and drivers, but also has a positive influence on Hong Kong society, fostering an environmentally friendly and safe atmosphere in roadside operations. By adopting the CVROSS, companies could save on costs and labour power for managing roadside activities, and thus business profitability could be improved.

## 6. Conclusions

Roadside activities, such as loading and unloading, negatively affect traffic situations if not kept under control. Hence, smart mobility is crucial for built-up areas in Hong Kong, aligning with smart-city development. This is especially the case where no real-time information about the roadside activities, occupancy and vacancy is accessible to the general public and road users. The CVROSS is a fully integrated solution, equipped with a set of wireless HD vision devices enabling image capture from the roadside, utilizing machine learning and supported by solar power, proposed as a real-time IoT-based system for surveillance of roadside loading and unloading bays. This can facilitate traffic and fleet management by implementing smart mobility, thus achieving a highly efficient road network. This paper contributes to the utilization of IoT technologies by developing the CVROSS in the Simulink and LabVIEW environments. From the set-up of parameters to vehicle and object recognition and matching with noise reduction, the calculation of parking gaps and available parking spaces and, lastly, to information visualization, the CVROSS, together with state-of-the-art IoT technologies, is able to provide road users with real-time roadside traffic information, such as roadside occupancy and vacancy, thereby enhancing the transparency of roadside activities. Various reports, e.g., daily reports, can also be produced for different parties via big data analysis. For example, logistics companies can optimize fleet schedules based on analytical information. In addition, fuzzy logic is applied in evaluating parking gaps and available parking spaces at the roadside, to establish decision support in roadside operations for enhancing evaluation accuracy and system flexibility. This is expected to help alleviate traffic congestion by reducing waiting times for loading and unloading activities and reducing costs of fuel and energy consumption by locating parking vacancies and preventing circling around the roads. The proposed CVROSS solution facilitates the development and management of a reliable and intelligent transport system in Hong Kong, resulting in the achievement of smart parking on the basis of smart mobility and smart transportation. Future efforts can be made to investigate, modify and realize the implementation of the CVROSS, to enhance the transparency of roadside activities. Ultimately, through the application of an IoT-based surveillance system for roadside loading and unloading bays, the development and management of a reliable and intelligent transport system in Hong Kong can be facilitated. In future work, the proposed system can be implemented in other regions affected by double-parking and busy roadside activities. In addition, automated and intelligent methods for formulating membership functions and fuzzy rules may be proposed.

## Figures and Tables

**Table 1:** Definitions of fuzzy classes and membership functions in the CVROSS.

Parameter/Unit	Range	Fuzzy Class	Membership Function	Type
*Input:*				
L_(*x,n*)_/m	[0, 18]	Short	[0, 5, 7]	trimf ^1^
		Medium	[5, 7, 11, 12]	trapmf ^2^
		Long	[11, 12, 18]	trimf ^1^
L_(*y,n*)_/m	[2, 4]	Narrow	[2, 2.5, 3]	trimf ^1^
		Medium	[2.5, 3, 3.5]	trimf ^1^
		Wide	[3, 3.5, 4]	trimf ^1^
*t_p_*/h	[0, 24]	Night hour	[0, 0, 6, 9]	trapmf ^2^
		Office hour	[6, 9, 15, 18]	trapmf ^2^
		Evening hour	[15, 18, 24, 24]	trapmf ^2^
*Output:*				
γ	[0, 1]	Slightly increased	[0, 0.33, 0.5]	trimf ^1^
		Substantially increased	[0.33, 0.5, 0.67]	trimf ^1^
		Significantly increased	[0.5, 0.67, 1]	trimf ^1^
*t_s_*/min	[0, 360]	Short	[0, 60, 120]	trimf ^1^
		Medium	[60, 120, 150, 210]	trapmf ^2^
		Long	[150, 210, 360]	trimf ^1^

Remarks: ^1^ trimf refers to the triangular shape of the membership functions; ^2^ trapmf refers to the trapezoid shape of the membership functions.

**Table 2:** Comparative analysis before and after using the CVROSS.

No.	Area	UoM ^a^	Before Using CVROSS	After Using CVROSS	% of Improvement
Perspectives from property management companies					
1	Severity of traffic congestion	Scale (1–10) ^b^	8.5	5.0	−41.2%
2	Severity of double parking	Scale (1–10) ^b^	9.7	6.5	−33.0%
3	Labour force on controlling roadside activities	people per shift	10	6	−40.0%
Perspectives from drivers and truckers					
1	Average fuel saving	Scale (1–10) ^b^	7.8	5.1	−34.6%
2	Average time to locate suitable parking space	min	18.2	8.8	−51.6%
3	Average driver satisfaction	Scale (1–10) ^b^	6.1	8.2	+34.4%

Notes: ^a^ UoM refers to unit of measurement; ^b^ Scale (1–10) refers to a Likert scale from 1 to 10, while 10 is the highest score in scale and 1 is the lowest score in scale.
